# Long-term efficient management of diabetic foot ulcer using simultaneous foot ulcer closure and surgical off-loading

**DOI:** 10.1016/j.jpra.2021.04.012

**Published:** 2021-06-07

**Authors:** Yuta Terabe, Nobuhito Kaneko, Keisuke Nakabayashi, Akihiro Matsui, Hiroshi Ando

**Affiliations:** Limb Salvage Center, Kasukabe Chuo General Hospital, 344-0063 midori5-9-4, Kasukabe, Saitama, Japan

**Keywords:** Surgical off-loading, Diabetic foot, Hard-to-heal foot ulcer, Simultaneous operation

## Abstract

Foot deformity is one of the causes of foot ulcers. Foot ulcers often recur due to foot deformity. Foot ulcer healing alone does not reduce the risk factor of foot ulcer recurrence. The majority of foot ulcer patients, most of whom are elderly, have foot deformities. This limits the chances of undergoing surgical off-loading following surgery. We present a case of diabetic foot ulcer (DFU) in which surgical off-loading was performed simultaneously with foot ulcer closure that did not recur for a period of 2 years following surgery. A 70-year-old diabetic male with a DFU presented to our hospital. He underwent surgical debridement followed by reconstruction surgery (stump plasty) simultaneous with surgical off-loading (metatarsal head resection).

The immediate postoperative period during which he used the off-loading device was uneventful. Following this, he used orthosis. After 2 years, the foot ulcer had not recurred, and he was able to independently perform his daily activities.

Simultaneous surgery (reconstructive surgery and surgical off-loading) is less likely to limit daily activities and can reduce the ulcer recurrence rate.

## Introduction

Foot deformity is one of the causes of foot ulcers.^1^ However, in patients with foot deformities, healing of a foot ulcer does not reduce the risk factor of foot ulcer recurrence.^2^ However, foot ulcers often recur due to foot deformity and the lifestyle of patients. The use of foot orthoses after the healing of foot ulcers prevents its recurrence.^3^ Hence, surgical off-loading is an effective method to prevent foot ulcer recurrence.^4^

Here, we present a patient with a hard-to-heal foot ulcer (diabetic foot ulcer, DFU) for whom surgical off-loading was performed concurrently with foot ulcer treatment.

### Case presentation

**Figure 1:**
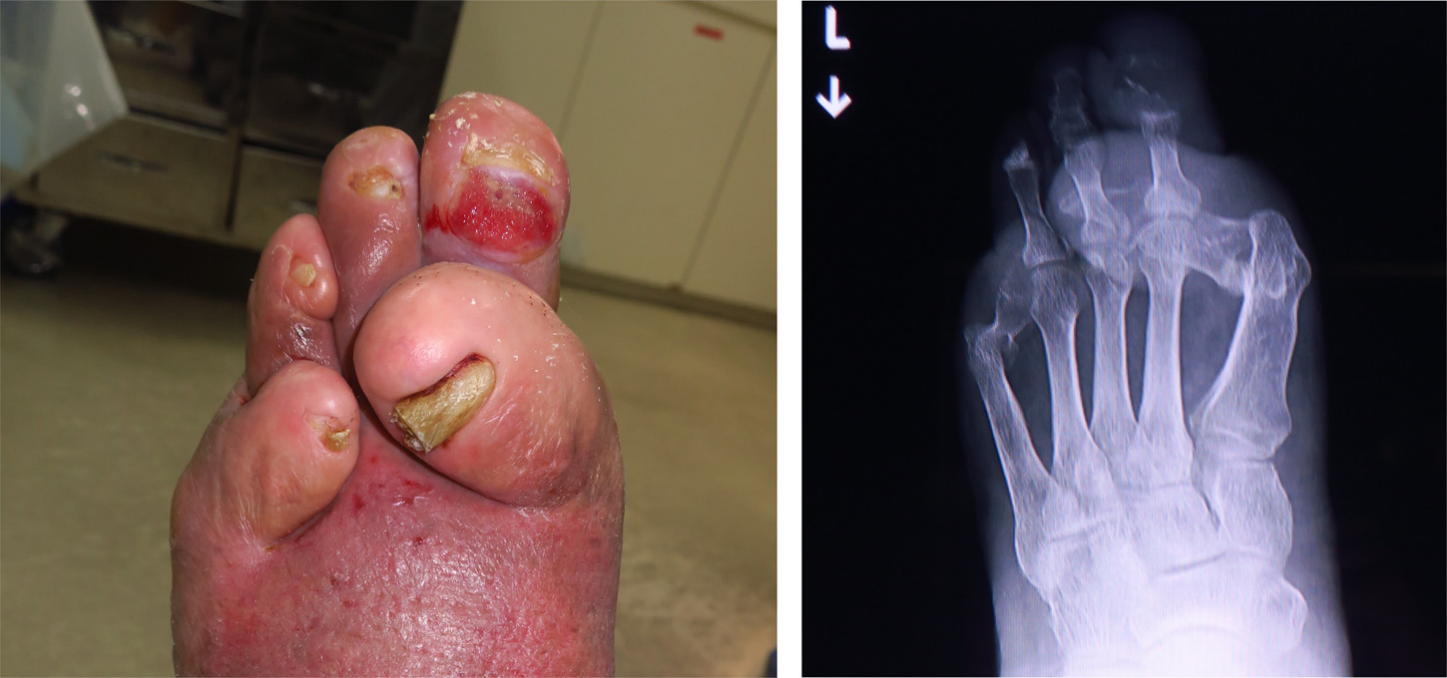
The left diabetic foot ulcer for the first time. (Left) A simple weight-bearing radiographic image of the left foot. (Right).

A 73-year-old patient had an uncontrolled DFU at the second toe for a period of six months (Figure 1-left). He had been diabetic for several decades, but he stopped the diabetic treatment by himself. On examination, he had no fever and was not weary. He had a local infection in the region of the second metatarsal head. This ulcer on his left foot exposed his second proximal interphalangeal joint (PIPJ). The skin around the ulcer was red and swollen, pointing towards the plantar surface of the second metatarsal phalangeal joints (MTPJ) from the ulcer. The ankle–brachial index was 1.18/1.26 (right/left), and skin perfusion pressure was 97/75 mmHg (dorsal/plantar). The second proximal phalangeal bone and middle phalangeal bone had evidence of destruction on observation of the left foot through a simple weight-bearing radiographic image (Figure 1-right). The MTPJ of the first and fifth digit was dislocated (Figure 1-right). The Wound, Ischemia, and foot Infection (WIfI) classification was W 2, I 1, fI 2, F 0.

**Figure 2:**
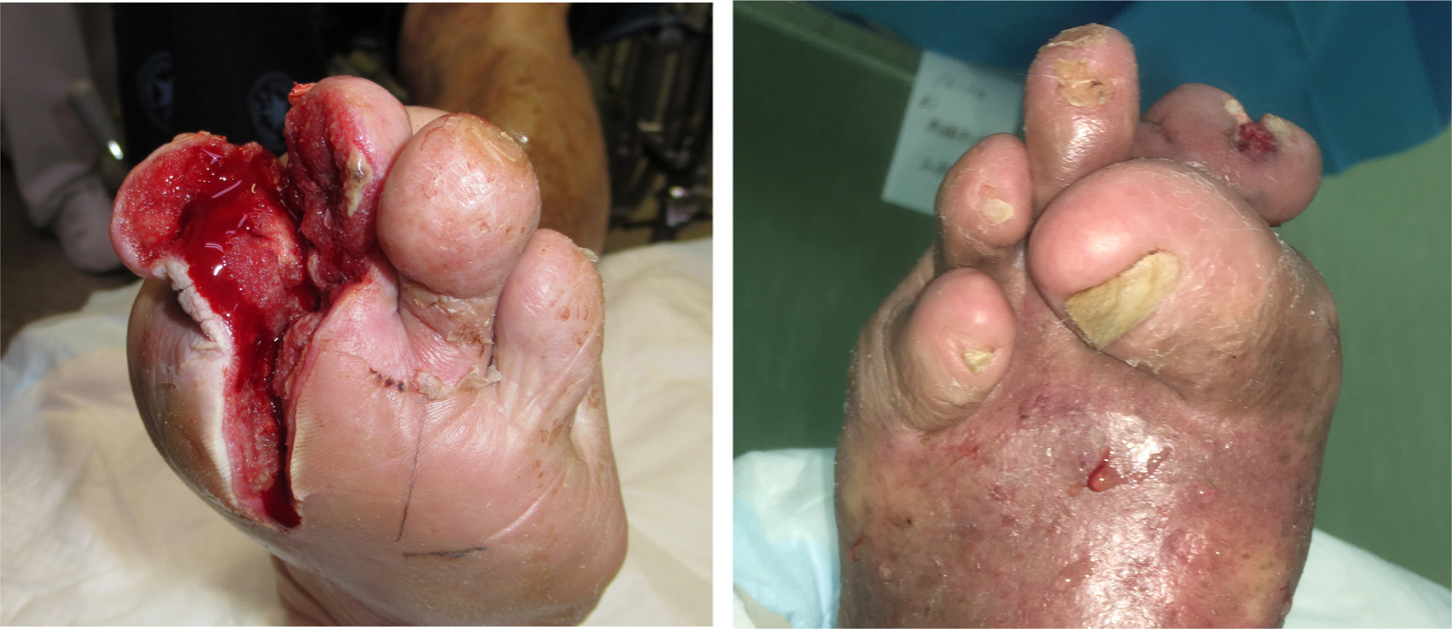
Post-debridement foot ulcer. (Left) Pre-operation foot ulcer. (Right).

**Figure 3:**
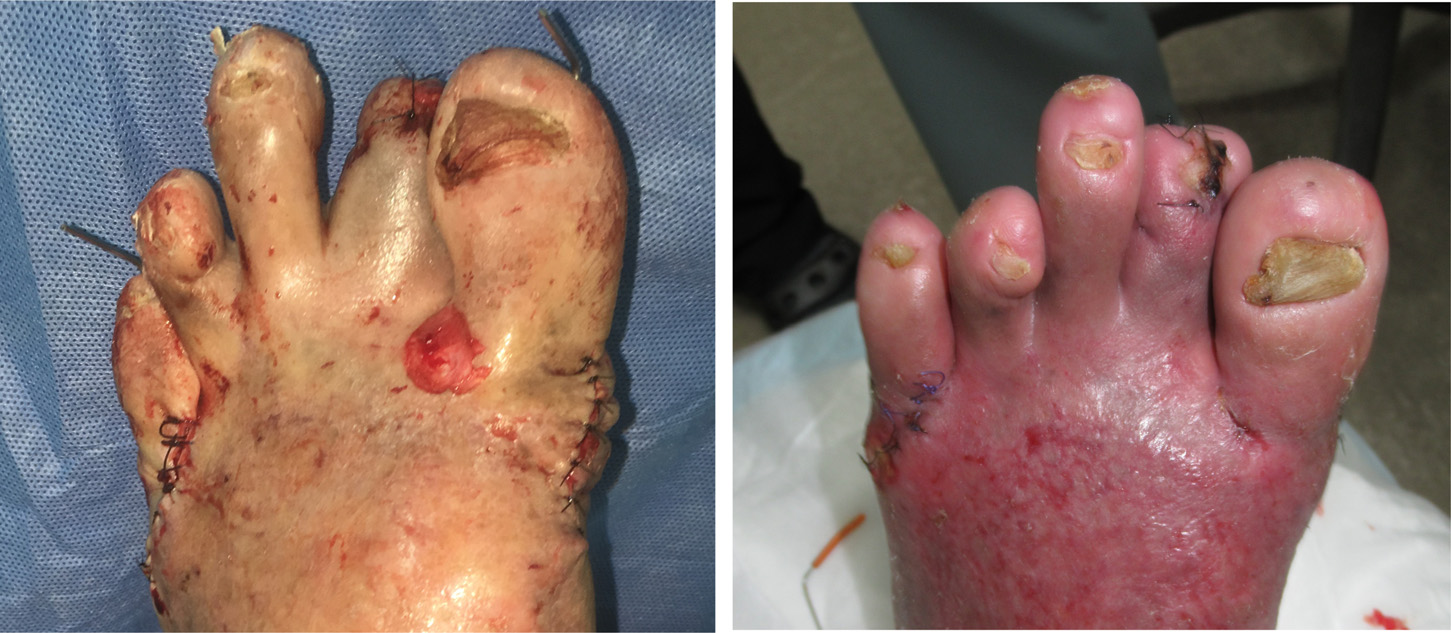
Pre-wound closure and surgical off-loading. (Left) Post-wound closure and surgical off-loading. (Right).

Therefore, a skin incision was made from the PIPJ to the MTPJ of the second ray at the outpatient department immediately (Figure 2-left). Two days post skin incision, skin redness and swelling reduced. Improvement was observed at the site of the local infection and as the foot ulcer underwent debridement. Post-debridement wound treatment continued for a period of two weeks until the ulcer was improved and replaced by a healthy granulation tissue (Figure 2-right). However, the MTPJ of the first and fifth digits of his left foot were still dislocated. The first toe rode the second toe, and the fifth toe rode the fourth toe at this point. Stump surgery of the second toe was therefore performed simultaneously with surgical off-loading. The surgical off-loading was done by resecting the first and fifth metatarsal heads (Figure 3-left). Two weeks following surgical off-loading, the surgical wound healed, and the patient was discharged from the hospital (Figure 3-right).

**Figure 4:**
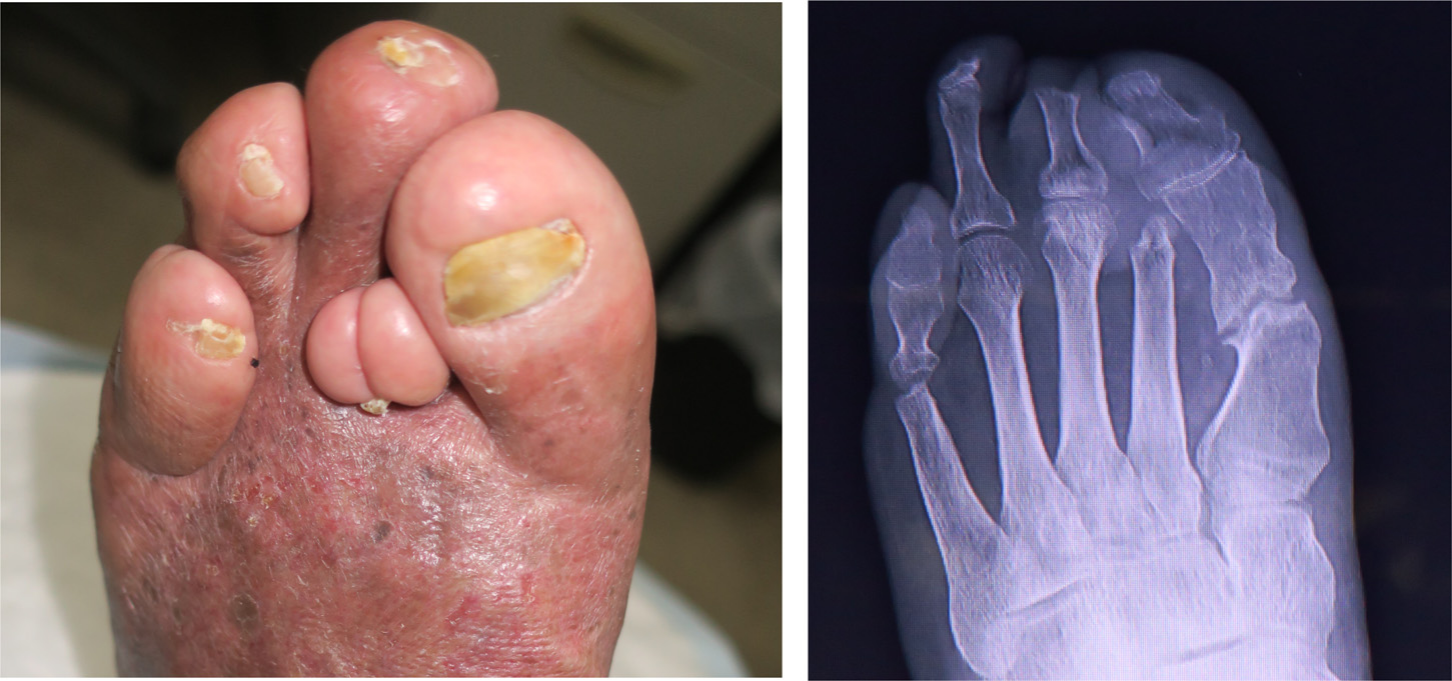
Left foot after 2 years. (Left) Left foot wound closure and surgical off-loading after 2 years. (Right).

On outpatient follow-up, he was still using a customised foot orthosis at his two-year appointment. He could walk independently, and the foot ulcer had not recurred (Figure 4-left). The corrected first and fifth ray was a little deformed. The second toe was shortened without the toe bone, and the first toe got in that space (Figure 4-right). The fifth toe had a mild varus deformity. The forefoot form was round shape and appeared to be due to his shoes. His soft tissue deformities resulted in his present foot form because there were no joints after metatarsal head resection. Actually, the motion of the first and fifth toe was flexible.

## Discussion

The patient, who presented with DFU, had an uneventful two-year postoperative period and could live independently.

Recent guidelines for diabetic foot management include surgical off-loading.^5^ Surgical off-loading is an operative technique to off-load a limb using a surgical procedure. It is primarily used to manage foot ulcers in selected patients, especially where other conservative off-loading options have failed. However, our management approach for this case differed from the typical methods. The surgical off-loading was performed to prevent new foot ulcer formation, and the existing ulcer was simultaneously treated. This is because older people have few chances to undergo surgical off-loading after their foot ulcers are cured. In addition, a resting postoperative period following surgical off-loading is required for the closure of the ulcer. Accordingly, the period for which the patient is confined to the hospital and in need of supportive care is considerably reduced.

## Conclusion

Surgical off-loading is recommended to be performed at a different time from wound treatment. However, when the foot deformity is severe and there is a clear risk of recurrence or it is difficult to perform surgical off-loading at another time, surgical off-loading should be performed simultaneously with wound healing. In the future, further studies are required through various case studies including randomized clinical trials.

Simultaneous surgical off-loading and closure of foot ulcers in hard-to-heal foot ulcers reduces the risk of ulcer recurrence.

## Declaration of Competing Interest

None
